# Shadow study: randomized comparison of clinic with video follow-up in glioma undergoing adjuvant temozolomide therapy

**DOI:** 10.2217/cns-2017-0024

**Published:** 2018-04-30

**Authors:** Vijay M Patil, Nikhil Pande, Arun Chandrasekharan, Chandrakanth M, Raees Tonse, Rahul Krishnatry, Jayant S Goda, Hollis Dsouza, Dilip Harindran Vallathol, Santam Chakraborty, Tejpal Gupta, Rakesh Jalali

**Affiliations:** 1 Department of Medical Oncology, Tata Memorial Hospital, Mumbai, India; 2 Department of Radiation Oncology, Tata Memorial Hospital, Mumbai, India

**Keywords:** clinic based, clinical, follow-up, glioma, surveillance, telephonic, video, visual

## Abstract

**Aim:**

This study was designed with a primary objective to study the rate of agreement in treatment plan and decisions between video follow-up (VF) and conventional clinic follow-up (CF).

**Patients & methods:**

Adult patients with intermediate- to high-grade glioma on adjuvant temozolomide (TMZ) with facilities for live video call were invited to participate in the study.

**Results:**

The concurrence in decision of administering TMZ between VF and CF was 100% (p < 0.00). The median cost incurred in VF was US$58.15 while that incurred in CF was US$131.23 (p < 0.00).

**Conclusion:**

VF can substitute CF during adjuvant TMZ administration (CTRI/2017/01/007626).

**Summary points**

- This study was designed with a primary objective to study the rate of agreement in treatment plan and decisions between video follow-up (VF) and conventional clinic follow-up (CF).
- A total of 112 patients were screened and 65 were accrued.
- The concurrence in decision of administering temozolomide between VF and CF was 100% (Cohen κ = 1.0, 95% CI: 1.0–1.0; p < 0.00).
- In concurrent medication domain (κ = 0.66, 95% CI: 0.04 −1; p < 0.00), imaging domain (κ = 1.0, 95% CI: 1.0–1.0; p < 0.00), rehabilitation domain (κ = 1.0, 95% CI: 1.0–1.0; p < 0.00) and molecular testing domain (κ = 0.65, 95% CI: 0.20–1; p < 0.00), the agreement was substantial.
- The patient satisfaction rate was 100% post-VF and was 98.5% post-CF.
- The median cost incurred by patient in VF was US$58.15 (IQR: 43.38–91.69) while that incurred in CF was US$131.23 (IQR: 68.8–256 [p < 0.00]).
- Hence it can be concluded that VF can replace CF if VF is logistically feasible.

Gliomas are the commonest primary brain malignancies seen in adults [1]. Many of these patients have significant physical or cognitive dysfunction [2], and need significant psychosocial support from the caregiving team [3]. In addition to traditional clinic visits, nurse-led telephonic follow-ups [4], and physician-led video follow-ups (VFs) have been explored as alternative follow-up approaches in such patients [5]. In patients with neurological symptoms and/or neurological deficits, a comprehensive video-based follow-up may have an advantage over telephonic follow-up as subjective and certain objective (neurological evaluations) can be performed via a live video call.

Patients with high- and intermediate-grade gliomas are treated with maximum safe resection followed by focal conformal radiation along with concurrent and adjuvant temozolomide (TMZ) [6]. During adjuvant TMZ therapy, grade 3 or 4 hematological toxic effects are seen only in 14% of patients [6]. In our own experience, the rate of grade 3–4 hematological toxicity was only 7% [7]. Whether these patients can be managed on VF and if the oncological outcomes of VF would be comparable to clinic follow-up (CF) remains an open question. However before undertaking such a study, we wanted to assess the safety and feasibility of subjecting these patients to such unconventional follow-up procedures. Hence, this study was designed with a primary objective to study the rate of agreement in treatment decision for administration of TMZ between VF and conventional CF.

## Methods

### Study

This was an investigator-initiated single-arm study conducted in the outpatient department of medical oncology (neuro-oncology disease management group) between January and March 2017. The study protocol was approved by the Institutional Ethics Committee and was registered with the Clinical Trial Registry of India (CTRI/2017/01/007626). The study was conducted in accordance with good clinical practice guidelines, the Declaration of Helsinki and institutional guidelines for human experimentation. The study was funded by Brain Tumor Foundation – India. The funding agency had no role in study design, data collection, analysis, interpretation or writing of the report.

Adult patients with histologically confirmed grade II–IV gliomas, on adjuvant TMZ, who had completed first two cycles or more, with adequate organ function and with means to participate in live video call were invited to participate in this study. Post-written informed consent, these patients underwent a routine CF. After completion of CF, the patients and their caregivers were provided a predetermined date and time for VF on 24th day (±3 days) and for the next CF on 28th day post first clinical visit (±5 days).

VF and the subsequent CFs were done by two different investigators. The six clinicians conducting follow-ups were divided into two groups. Each patient was allotted to the group according to a computer generated random number table. Simple randomization was used, and group allocation was done by a neutral person situated in a different city. To avoid inter-clinician bias, common clinical scenarios were discussed and a consensus was arrived on decision making. Consensus guidelines were adopted for the decision on blood parameter values required for administering new cycle of TMZ [8]. Interclinician agreement prior to the start of the study for clinic-based follow-up was 100% agreement on all domains except the need for rehabilitation domain, where the agreement was 90%. In VF, however, the agreement was 100% on all domains.

During the video follow-up session, the physician enquired about new symptoms (headache, vomiting, imbalance, etc.) and noted the status of symptoms reported in previous visit. A subjective assessment of the cognitive function (orientation in time, place and person), cranial nerve function (vision, diplopia, chewing, swallowing, etc.), motor function (difficulty in lifting arms, moving fingers, etc.), sensory function and drug-associated side effects was performed.

Cognitive dysfunctions and drug-associated side effects were recorded as a binary dichotomous outcome (present or absent). For other domains, the response was recorded as trichotomous outcome (improved/same/deteriorated with respect to the previous visit).

The objective assessment consisted of recording the presence of intact cranial nerve (eye movements, facial movements, chewing, shoulder shrugging, etc.) and motor functions (upper and lower limb movements). The blood counts were documented.

After this assessment, the decision regarding continuation of TMZ (yes/no), supportive medications (yes/no), need for imaging (yes/no), need for molecular testing (yes/no) and need for rehabilitation (yes/no) were documented in VF proforma and conveyed to the patient and or caregivers. They were informed that the plan was tentative and the plan post-CF would be the final plan. The investigators were encouraged to document the problems faced during VF. Post-VF, SMS (short message service) enquiring about the patient's satisfaction with the VF. Four options (‘extremely dissatisfied’, ‘somewhat dissatisfied’, ‘somewhat satisfied’ and ‘extremely satisfied’) were provided with a numerical code. Patients or caregivers were asked to provide their response within 48 h. As no reply came in 48 h, a repeat SMS was sent and a reminder call was made. In spite of these efforts, if patient did not reply then it was assumed that patient was not willing to provide this information.

The CF was done 4 days post-VF and the investigator performing the CF was blinded to the VF plan.

The subjective and objective evaluations were similar to those done in VF except that sensory examination was performed. The blood counts were noted. Post this assessment, the decision regarding continuation of TMZ (yes/no), supportive medications (yes/no), any need for imaging (yes/no), need for molecular testing (yes/no), need for rehabilitation (yes/no) and next visit plan was documented in CF proforma and conveyed to the patients or caregivers. In addition, the direct and indirect costs incurred during the visit and video call were captured. The patients satisfaction status with the CF was documented again using a SMS. After completion of CF, patients were deemed to have completed the study interventions and the further management was in accordance with institutional standards.

### Statistical analysis

The decisions in VF and CF were compared on five domains. These were decisions of administration of TMZ (primary end point), decision of administration of concomitant supportive medications, decision on requirement of brain imaging, decision on need for any molecular testing and decision on need for physiotherapy. The VF plan was deemed to have concurred with the CF plan if the dose of administered TMZ was the same (±10%). For concomitant medications domain, the VF final plan was considered to have concurred with the CF plan if the number and type of antiepileptic supportive medicines were the same. Agreement analysis included calculation of Cohen's kappa coefficient (κ) for each domain, which were then interpreted as per Landis and Koch (κ score of ≥ 0.6 indicative of good agreement) [9].

The comparison between satisfaction rates was done by the Fisher's exact test. For the purpose of analysis, the responses were categorized into two categories – satisfied and dissatisfied based on the response provided through the SMS. A p-value of <0.05 was considered as statistically significant.

The cost of VF included the cost of video call, cost of complete hemogram, cost of TMZ and supportive medications and a hypothetical cost of courier of medications to the patient's home (cost at the time study for delivery of 100 g courier was taken for uniformity). The cost of CF included the cost of travel of patients and attendants, complete hemogram, TMZ, supportive medications and loss of pay incurred by the patient and attendant. The details of cost calculation are shown in Supplementary Appendix. The aggregate cost distribution was tested by Mann–Whitney–Wilcoxon test.

### Sample size calculation

The sample size was calculated for agreement analysis assuming a κ coefficient of 0.9, with a one-sided confidence interval for lower limit of 0.6, for 2 raters, binary outcome and α of 0.05. The sample size was 52. Assuming a lost to follow-up rate of 20%, the total sample size obtained was 65. The κ of 0.9 was chosen as it is considered as near perfect agreement. The lower limit of κ was chosen as 0.6 as κ above this value signified substantial agreement [9].

## Results

### Baseline details

The median age was 41 years (interquartile range [IQR]: 33–49 years) and males constituted 73.8% of patients. The diagnosis were glioblastoma in 24 patients (36.9%), anaplastic astrocytoma in 15 patients (23.1%), astrocytoma grade 2 in four patients (6.2%), oligodendroglioma in five patients (7.7%) and oligodendroglioma grade 3 in 17 patients (26.2%). The median number of adjuvant cycles completed before enrollment in the study were six (IQR: 4–8). The details of baseline details are provided in Table 1.

**Table 1.** **Baseline details of enrolled patients.**

**Variable**	**Value**
Median age (years)	41 (IQR: 33–49)

Gender: – Male – Female	48 17

ECOG performance status: – 0 – 1 – 2	2 (3.1%) 61 (93.8%) 2 (3.1%)

Neurological performance status: – 0 – 1 – 2	41 (63.1%) 22 (33.8%) 02 (3.1%)

Tumor details: – Astrocytoma grade – 2 Anaplastic astrocytoma – Glioblastoma – Oligodendroglioma grade 2 – Oligodendroglioma grade 3	4 (6.2%) 15 (23.1%) 24 (36.9%) 5 (7.7%) 17 (26.2%)

*IDH* mutation status: – Mutated – Wild-type – Unknown	43 (66.2%) 19 (29.2%) 03 (4.6%)

*MGMT* methylation status: – Methylated – Wild-type – Unknown	18 (7.7%) 13 (20.0%) 34 (52.3%)

Treatment details: – Median number of cycles of TMZ before enrollment	6 (IQR: 3–8)

Median number of antiseizure medications ongoing	1 (IQR: 1–2)

Antiseizure medications: – Phenytoin sodium – Sodium valproate – Carbamazepine – Levetiracetam – Clobazam – Oxcarbazepine – Phenobarbitone – Lacosamide	31 (50.7%) 1 (1.5%) 1 (1.5%) 46 (70.8%) 7 (10.8%) 1 (1.5%) 1 (1.5%) 1 (1.5%)

Need for steroid	1 (1.5%)

Native province: – Maharashtra – Bihar – Chhattisgarh – Gujarat – Jharkhand – Madhya Pradesh – Orissa – Rajasthan – Uttar Pradesh – West Bengal	40 (61.5%) 1 (1.5%) 1 (1.5%) 1 (1.5%) 1 (1.5%) 3 (4.6%) 1 (1.5%) 4 (6.2%) 7 (10.8%) 6 (9.2%)

Network service providers: – Airtel – Idea Cellular – Reliance Jio – Bharat Sanchar Nigam Limited – Vodafone – Others	19 (29.2%) 7 (10.8%) 10 (15.4%) 4 (6.2%) 23 (35.4%) 2 (3.1%)

Education: – Illiterate – Primary schooling – Secondary schooling – Higher secondary schooling – Graduation – Postgraduation	5 (7.7%) 9 (13.8%) 22 (33.8%) 8 (12.3%) 16 (24.6%) 5 (7.7%)

Median yearly family income (US$)	923.08 (IQR 0–2500)

ECOG: Eastern Cooperative Oncology Group; IQR: Interquartile range; TMZ: Temozolomide.

### Decision domains

The concurrence in terms of decision of administering TMZ between VF and CCF was 100% (κ = 1.0 95% CI 1.0–1.0, p < 0.000). There was a substantial concurrence in decision on concomitant medications between VF and CF (κ = 0.660, p < 0.000). There was a discrepancy in decision of one patient, who had an episode of absence seizures post-VF before CF (Table 2). Hence an additional antiepileptic was added during his CF. The concurrence on need for imaging and physiotherapy was 100% (κ = 1, p < 0.000). In the need for molecular testing domain, discrepancy was seen in two patients, leading to a substantial agreement (κ = 0.652, p < 0.000). These two patients required additional molecular testing which was missed on VF.

**Table 2.** **Details of agreement analysis in different domains.**

**Domains**	**Number of patients in whom discrepancy was found**	**Cohen's κ (95% CI)**	**p-value**
Temozolomide administration decision	–	1	<0.000

Antiepileptic administration decision	1	0.660 (0.04–1)	<0.000

Decision on requirement of brain imaging	–	1	<0.000

Decision on need for any molecular testing	2	0.652(0.20–1)	<0.000

Decision on need for physiotherapy	–	1	<0.000

### Satisfaction rate

The satisfaction status postvideo follow-up was somewhat satisfied in 13 patients (20%) and extremely satisfied in 52 patients (80%). While the same after CF was somewhat satisfied in eight patients (12.3%), extremely satisfied in 56 patients (86.2%) and somewhat dissatisfied in one patient (1.5%). All patients expressed their satisfaction with VF (n = 65, 100%) while 64 (98.5%) were satisfied with CF. As all patients undergoing VF were satisfied, no measure of association could be computed.

### Cost analysis

**Figure 1.**
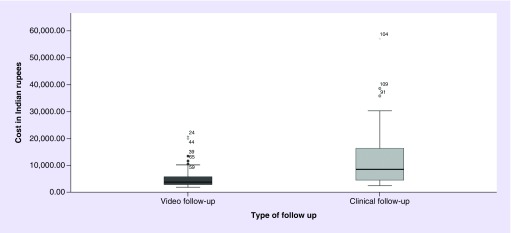
**Total cost versus the type of follow-up.** The box plot depicts the median and interquartile range for cost incurred in each type of follow-up.

**Figure 2.**
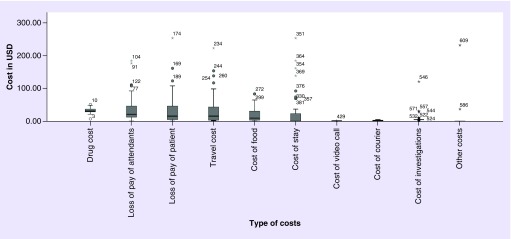
**Types of costs incurred under each heading are depicted.** The box plot under each heading depicts the median and interquartile range for cost incurred.

The median cost incurred in VF was US$58.15 (IQR 43.38–91.69) while that incurred in CF was US$131.23 (IQR 68.8–256, p < 0.000) (Figure 1). The details of the cost incurred are shown in Figure 2.

### Issues during video calls

The number of video calls required to complete the interview was one in 28 patients (43.1%), two-to-three in 25 patients (38.5%) and greater than three in 12 patients (1.8%). Network issues during video calls were encountered in 33 interviews (50.8%). The issues included frequent drops in 28 patients (43.1%) and poor connectivity in five patients (7.7%). The application used for video call were WhatsApp in 48 patients (73.8%) and IMO in 17 patients (26.2%) respectively. During video calls the application was not updated in 31 patients (47.7%). These patients were then informed on audio call to update the applications and then video call was performed. The video and voice status was found satisfactory by the investigators doing VF in 47 (72.3%) and 59 patients (90.8%), respectively.

## Discussion

The study demonstrates that the decisions regarding administration of chemotherapy can be safely taken with live video calls. Thus, CF during adjuvant TMZ administration may be safely substituted by VF. Perfect agreement in the decisions taken during VF and CF was seen in all domains except for the molecular testing domain. Our institution is a premier tertiary care cancer center and nearly 50% of the patients seen in our outpatient department are referrals postsurgery from another center. As a routine, slides are reviewed for confirmation of diagnosis but paraffin blocks are routinely not available with the patients. Hence, although molecular testing is requested in all patients, logistic issues in acquisition of blocks may lead to a delay in molecular testing. Therefore, during the adjuvant treatment, we stress on the need for molecular testing if it had not been performed. Physicians during VF failed to address the question of molecular testing in two patients.

It was assumed by the investigators that patients might have higher satisfaction rates when they have a CF, as seen in other studies from India [10,11]. It was believed that even if the decisions were similar between the two types of follow-up, a high degree of dissatisfaction with VF would reduce its acceptability in practice. These results demonstrate that VF had similar satisfaction rates as CF. The investigators noted a high level of enthusiasm and acceptance on the part of the participants for the VF during the video calls. CF in glioma patients with neurological deficits is cumbersome and results in financial, mental and physical hardships in both patients and their caregivers [12,13], which can be avoided by VF. Multiple studies have questioned the need for clinic-based follow-up in cancer patients [14]. A large UK follow-up practice analysis revealed that research is needed to determine what preferences patients and families have for follow-up and what their level of satisfaction is with these methods [15].

Another important finding of the study was lower cost incurred in conducting VF than CCF. This highlights the considerable amount of financial loss for the subject or for the province, in case of province sponsors the healthcare, which could be avoided if VF is done. The costs of CF were inflated mainly because of the travel and accommodation costs in the city of Mumbai (Figure 1B), as nearly 50% of the patients in our study hailed from outside Mumbai. Presence of limited cancer centers is an issue even in high-income countries and therefore VF might help circumvent the issue [16].

Out of 112 screened patients, only six patients could not be enrolled because of the lack of a smart phone device. All VF were possible with applications using locally available telephonic networks. VF in some patients had a few technical glitches which were easily circumvented by careful planning. Network issues lead to multiple calls due to frequent call drops. These issues would hopefully improve with time as more advanced wireless telephonic technology gets adopted across the country [17]. This situation is rapidly going to change as cheaper smartphones that are 3G–4G enabled are being pumped into the market.

Cancer centers are sparsely distributed and in many instances patients have to travel long distance for cancer care [18]. Such travels in patients with glioma with neurological deficit is difficult. Hence, we wanted to see whether video follow-up can be used in this situation. However before taking such a randomized study, we wanted to be sure that the decisions we take on video follow-up and CF are concordant and hence we did this study. The results of this study have reassured us and we are now going ahead with a randomized study SHADOW-2 to compare whether video follow-up can completely replace CF in glioma patients postadjuvant chemoradiation.

The study has its own strengths and limitations. This is the first study comparing CF versus VF in a therapeutic setting. Multiple studies have compared CF with voice calling in a post-treatment follow-up or surveillance setting. The investigators who did CF and VF were trained expert oncologists. They had all agreed upon an algorithm for management and underwent a quality check before the start of the study. The accessories and networks used for the study were commonly available with the patients and hence, no extra resources were utilized for the VFs. The duration between the VF and CF was short (∼4 days), thus ensuring that there were not any major changes in the clinical scenarios in between the two follow-ups. The primary limitation of the study was that it was a single center study. In addition, this was done after the second cycle of adjuvant TMZ so the applicability of this follow-up method during earlier cycles remains to be studied.

## Conclusion

The decisions taken regarding administration of adjuvant TMZ were similar between VF and CF. Hence, it may be possible to substitute CF with VF during adjuvant TMZ administration whenever it is logistically feasible for doing a video call, as it is practical and economical.

## Future perspective

VF might be in naive state at present but further research will enable us to do online consultations. We feel that in 5–10 years from now this would be the primary mode of consultations.
