# Supplementary material for: Shadow study: randomized comparison of clinic with video follow-up in glioma undergoing adjuvant temozolomide therapy
Source: CNS Oncol. 2018 Apr 30;7(2):CNS14. doi: 10.2217/cns-2017-0024 (PMC5977274; doi:10.2217/cns-2017-0024)
Supplement: Supplementary file 1 [file cns-07-14-s1.docx]

## Cost sheet

| No | Item | Cost per entity | Total cost per section |
| --- | --- | --- | --- |
| 1 | Cost of medications inclusive of temozolomide, supportive medications ( steroids, antiemetics and antiepileptics) and other ongoing medications  *Method : Will be obtained from the TMH prescription record at that visit. In case of medications acquired from outside pharmacy the MRP will be considered* | Cost of TMZ:  Cost of supportive medications :  Cost of other medications : |  |
| 2 | Cost of loss of pay of relative  *Method : The pay of relative will be enquired from him/her and would be divided by 30 for getting a day's pay. In case of female house wife the pay per day will be assumed as 1000 rs. If more than one relative accompanies the cost considered will be for each relative/.* | Per day pay (P1)  Number of days lost  Total cost = P1 X number of days lost  In case of more relatives ( > 1) use the below formula  Total cost = ( ∑ P from 1 to i ) X numbers of days lost  Summation stands for addition of each day pay of each relative. For ex if 3 relatives are present than ∑ P from 1 to i = P1+P2+P3 where P1 P2 & P3 are pays per day of each relative . |  |
| 3 | Cost of loss of pay of patient if working  *Method : To enquire whether patient has joined duty or is working . If not then assumed as 0. If yes then pay will be enquired from him/ her and would be divided by 30 for getting a day's pay. In case of female house wife the pay per day will be assumed as 1000 rs* | Per day pay  Number of days lost |  |
| 4 | Cost of travel  *Method : Will enquire about place of stay. Cost on travel from house still hospital will be enquired and multiplied by number of persons travelling ( inclusive of patient).* | One sided travel cost (T1) :  Number of persons travelling ( N1):  Total travel cost + T1X N1 X 2 = |  |
| 5 | Cost of food  *Method : Cost per meal during travel and stay in mumbai will be enquired. Total cost for meals will be calculated as cost per meal* | Average Cost per meal (M1)  Total number of meals per person (M2)  Total cost = M1 X M2 X number of persons (N1) |  |
| 6 | Cost of staying in mumbai  *Method : Cost for entire duration of stay in mumbai for patient with relatives would be documented. In case of stay in relatives house or at footpath. A arbitrary cost of 1500 Rs per 2 patients per night would be used for calculation* | Numbers of room -nights (C1)  Cost per room night (C2)  Total cost = C1XC2 |  |
| 7 | Cost of Video call  Method : The video call will be made by the investigators. The balance before and after the video call be checked and the exact cost of call will be noted. |  |  |
| 8 | Hypothetical cost of courier  *Method : The patient's address will be noted. Online from DTDC website the cost of doing a DTDC Lite air courier with 300 g weight will be noted* |  |  |
| 9 | Cost of investigations  Cost of blood test for the visit |  |  |
| 10 | Other cost |  |  |
| Total cost | | |  |
| Total cost for CCF = 1+2+3+4+5+6+9+/-10 | | |  |
| Total cost of VF = 1+3+7+8+9+/-10 | | |  |
